# GlycReSoft: A Software Package for Automated Recognition of Glycans from LC/MS Data

**DOI:** 10.1371/journal.pone.0045474

**Published:** 2012-09-26

**Authors:** Evan Maxwell, Yan Tan, Yuxiang Tan, Han Hu, Gary Benson, Konstantin Aizikov, Shannon Conley, Gregory O. Staples, Gordon W. Slysz, Richard D. Smith, Joseph Zaia

**Affiliations:** 1 Bioinformatics Program, Center for Biomedical Mass Spectrometry, Department, of Biochemistry, Boston University, Boston, Massachusetts, United States of America; 2 Center for Biomedical Mass Spectrometry, Department, of Biochemistry, Boston University, Boston, Massachusetts, United States of America; 3 Genome Technology Branch, National Human Genome Research Institute, National Institutes of Health, Bethesda, Maryland, United States of America; 4 Biological Sciences Division and Environmental Molecular Sciences Laboratory, Pacific Northwest National Laboratory, Richland, Washington, United States of America; 5 Broad Institute of Massachusetts Institute of Technology and Harvard, Cambridge, Massachusetts, United States of America; University of Patras, Greece

## Abstract

Glycosylation modifies the physicochemical properties and protein binding functions of glycoconjugates. These modifications are biosynthesized in the endoplasmic reticulum and Golgi apparatus by a series of enzymatic transformations that are under complex control. As a result, mature glycans on a given site are heterogeneous mixtures of glycoforms. This gives rise to a spectrum of adhesive properties that strongly influences interactions with binding partners and resultant biological effects. In order to understand the roles glycosylation plays in normal and disease processes, efficient structural analysis tools are necessary. In the field of glycomics, liquid chromatography/mass spectrometry (LC/MS) is used to profile the glycans present in a given sample. This technology enables comparison of glycan compositions and abundances among different biological samples, i.e. normal versus disease, normal versus mutant, etc. Manual analysis of the glycan profiling LC/MS data is extremely time-consuming and efficient software tools are needed to eliminate this bottleneck. In this work, we have developed a tool to computationally model LC/MS data to enable efficient profiling of glycans. Using LC/MS data deconvoluted by Decon2LS/DeconTools, we built a list of unique neutral masses corresponding to candidate glycan compositions summarized over their various charge states, adducts and range of elution times. Our work aims to provide confident identification of true compounds in complex data sets that are not amenable to manual interpretation. This capability is an essential part of glycomics work flows. We demonstrate this tool, GlycReSoft, using an LC/MS dataset on tissue derived heparan sulfate oligosaccharides. The software, code and a test data set are publically archived under an open source license.

## Introduction

The development of technology for characterization of released glycans is driven by biological relevance with the goal of providing detailed glycan structure as targets for synthesis, biomarker, and therapeutic development. Towards these ends, the determination of glycomics MS profiles is an essential step that enables investigators to identify glycan compositions and abundances. With this profiling information, investigators then designate glycan compositions for further purification and detailed tandem mass spectrometric analysis. LC/MS is particularly useful for glycomics profiling because the chromatography dimension enables robust and sensitive instrument performance. The addition of a chromatography dimension results in greatly improved ability to sample the complete set of glycans present. Chromatographic separation also minimizes the extent of ion suppression and thereby maximizes dynamic range.

Unfortunately, interpretation of the LC/MS profiling datasets requires considerably more time than does data acquisition, severely limiting glycomics workflows. Ions from a given glycan composition are typically multiply charged and therefore require deconvolution in order to reduce the redundancy caused by charge. In addition, glycan ions may be observed in more than one adducted form in the mass spectra. Therefore, it is necessary to combine charge states and adducted forms for a given glycan composition in order to produce definitive neutral masses and the corresponding accurate abundances. These characteristics of the LC/MS data render manual interpretation very time consuming.

Over the past decade, bioinformatics software specific to the needs of glycan and glycoconjugates has been developed. The Glycomod program [1] determines glycan compositions from mass spectral data. This program is available through the ExPASy portal for assignment of released glycan compositions or glycans attached to a protein or peptide. The input is a glycan mass list and the program is not applicable to automated data analysis of LC/MS datasets. Glycoworkbench is a software tool that contains features for structure drawing, assignment of glycan compositions from mass spectra, and assignment of product ions from tandem mass spectra [2]. This tool is used for piecemeal spectral interpretation; it is not applicable to automated data analysis. Cartoonist is a tool for annotating matrix assisted laser desorption/ionization (MALDI) mass spectral peaks of permethylated *N*-glycans [3]. This tool assigns plausible glycan structures to MALDI ions based on biosynthetic rules. The tool does not deconvolute, is not automated, and is not amenable to analysis of LC/MS datasets.

Although glycans may be regarded as metabolites, they are larger in mass than considered in typical metabolomics analyses, in which compounds are less than 1000 Da and produce singly charged ions. As a result, metabolomics algorithms are not applicable to glycomics. For glycomics LC/MS, high resolution, high mass accuracy MS is necessary in order to define glycan *m/z* values and charge states accurately; thus deconvolution is an essential data processing step. In addition, glycans as a compound class range from neutral to acidic, and use of negative polarity MS is often recommended. Decon2LS is an open-source software package designed for automated processing of high resolution mass spectral data [4]. Decon2LS uses a form of the THRASH (Thorough High Resolution Analysis of Spectra by Horn) algorithm [5] to de-isotope mass spectra. Although originally designed for peptides, this tool enables the user to adjust average residue elemental composition (polyaveragine) to values appropriate for glycomics LC/MS data. It reads native data formats from a number of mass spectrometer manufacturers, thus eliminating the need to convert to a public data format. Glycomics LC/MS data are often acquired using negative polarity, and Decon2LS processes the negative charge states correctly.

Glycosaminoglycans (GAGs) are a class of polysaccharides that occur on proteins on all animal cell surfaces and in the surrounding extracellular matrixes. These linear polysaccharides mediate binding to many growth factor families and influence cellular responses to environmental stimuli. Heparan sulfate (HS), a member of the GAG compound class, is characterized by its variations in sulfation patterns. Its expression is required for embryonic development [6] and for normal functioning of every adult physiological system [7]. The structure of HS depends on biosynthetic enzymes present in the Golgi apparatus, the levels of which are under complex regulation [7]. As a result, the structure of HS in biological systems varies dynamically depending on spatial and temporal factors. Cells modulate the manner in which they respond to growth factor stimuli by altering the structure of HS on their surfaces and in the surrounding extracellular matrices. Thus, the structure of HS depends on the cell type and the context in which it is growing.

Methods for structural characterization are essential for developing an understanding of the mechanisms whereby HS mediates biological functions and exploiting this information in the service of human health. A key step in this process is comparative profiling of HS from different biological sources from LC/MS data. Methods based on LC/MS have been used for this purpose [8], [9], [10], [11], [12], [13], [14], [15], [16], [17], [18].

In previous work, we developed a software program (Manatee) that alleviates this bottleneck by extracting lists of targeted compounds from LC/MS data [19]. While Manatee allows for rapid extraction of HS compositions and abundances from the raw data, it does not provide a means of noise reduction or confidence measurement. Decon2LS produces a list of deconvoluted masses and abundances for LC/MS datasets. In this work, we describe a new algorithm (GlycReSoft) that provides both rapid extraction of glycan compositions (and their abundances) from the Decon2LS output as well as a means of scoring the results to facilitate confident use of glycomics LC/MS data. GlycReSoft implements supervised and unsupervised scoring methods that enable assignment of peaks to both known and unknown glycan compositions. The capability of GlycReSoft was tested using LC/MS datasets generated on tissue derived HS.

## Materials and Methods

### Data Preparation and Preprocessing

LC/MS data were acquired on bovine organ HS samples using an Agilent Technologies 6520 QTOF mass spectrometer using a chip interface as described [9], [10]. Briefly, HS samples were digested exhaustively using heparin lyase III. The oligosaccharides were analyzed using a chromatography chip (Agilent Technologies, Santa Clara, CA) packed with amide-silica hydrophilic interaction chromatography (HILIC) stationary phase [10]. The HS oligosaccharides were analyzed using negative polarity MS detection. All LC/MS data were processed using the DeconTools [20] version of the Decon2LS program [4]. The averagine formula was set to C_6_ H_11.375_ N_1.125_ O_9.5_S_1.5_. The DeconTools parameters, output files, the GlyReSoft compiled software, source code, and user instructions have been publicly archived (http://code.google.com/p/glycresoft/downloads/list).

GlycReSoft is in principle applicable to any compound class from LC/MS data deconvoluted using DeconTools. Users interested in glycan classes other than heparan sulfate are advised to estimate the average monosaccharide elemental composition and use this as the averagine formula with DeconTools.

### GlycReSoft Algorithm

**Figure 1:**
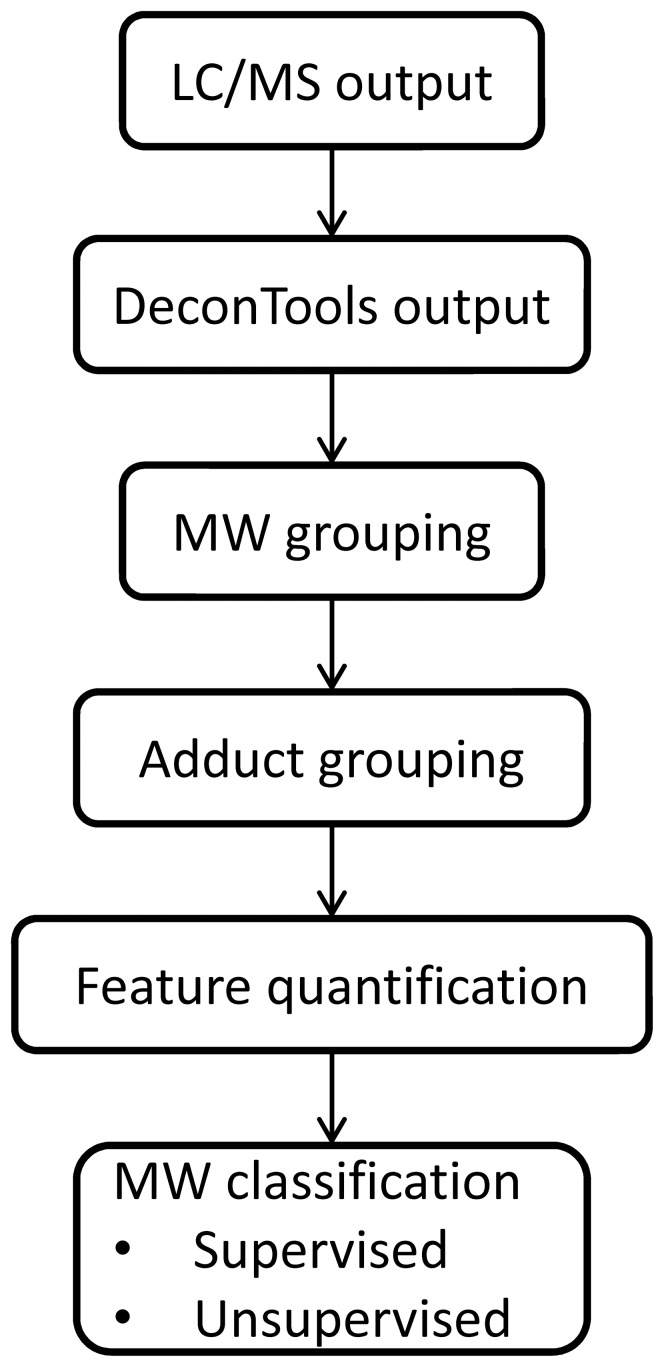
GlycReSoft workflow. The LC/MS data consist of three dimensions (*m/z*, abundance and time). The data are processed into lists of neutral masses and abundances using Decon2LS/DeconTools. GlycResoft combines the raw neutral masses into compounds, correcting for mass spectrometric adducts. The program scores the data, generates a list of candidate glycan compositions, and matches these against the compound list.

GlycReSoft predicts and scores candidate compounds based on quantitative features derived from calculations made by Decon2LS/DeconTools for each LC/MS data file. This scoring provides the ability to rank candidate compounds by perceived confidence, thereby distinguishing true glycans from noise. The main steps of GlycReSoft are represented in Figure 1. First, raw peaks are grouped in the mass dimension (MW grouping) across all time points (scans) into representative masses based on a user-defined tolerance range measured in parts per million (ppm), where 1 ppm = ΔM/M×10^6^. Second, GlycReSoft allows for grouping of compounds in multiple adduct forms (adduct grouping) by identifying representative masses that differ by a user-provided molecular weight or chemical formula corresponding to a known chemical adduct observed in LC/MS. Third, the various measurements made by Decon2LS/DeconTools are accumulated based on the previous grouping steps to yield a summarized list of unique MWs representing candidate compounds. Finally, the summarized list is scored, using either supervised or unsupervised learning methods that utilize the quantitative features of Decon2LS/DeconTools, to produce a ranking of candidate compounds.

**Figure 2:**
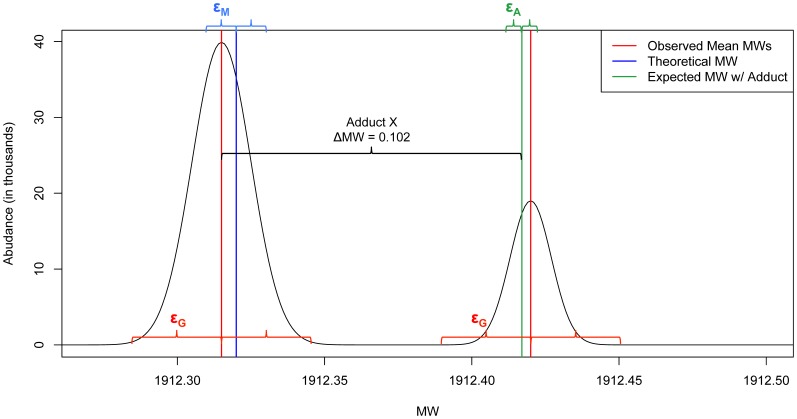
Mass tolerances specified in GlycReSoft. Note that in the GlycReSoft GUI 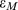 is referred to as match error, 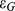 as grouping error, and 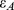 as shift adduct tolerance.

The first grouping over mass and time takes into account the instrumental noise, often at low abundance, that is observed in the LC/MS data and deconvoluted by Decon2LS/DeconTools. GlycReSoft achieves a locally optimal grouping by binning molecular weights greedily in descending order by peak abundance. Bins are created and centered at the first encountered molecular weight (*MW_0_*) and span the range of 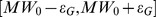, where 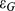 is the user-defined *grouping tolerance* in ppm (Figure 2). After all of the Decon2LS/DeconTools output is grouped, the abundance-weighted mean for each bin becomes the representative MW for that candidate compound group.

The second grouping over adduct states is implemented as a log-linear search for pairs of representative masses from the first grouping that differ by the user-defined molecular weight of the adduct of interest, denoted here by Δ*MW*. In practice, representative masses are investigated in ascending order where adducted forms of a candidate compound with molecular weight *MW_0_* are identified if their molecular weight falls in the range of 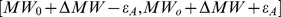, where 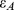 is the user-defined *adduct tolerance* (Figure 2). A series of candidate compound groups are then collapsed into a single candidate compound representing multiple adduct forms. For each series of candidate compounds, the cumulative abundance is summed across all adduct forms and the form with the largest abundance becomes the new representative MW for the group.

The two grouping steps result in a list of unique masses representing candidate compounds. GlycReSoft allows the user to filter out low quality candidates, if desired, through the use of optional thresholding parameters, including upper and lower MW bounds and thresholds for minimum abundance and number of detected scans. Most LC/MS users have prior knowledge concerning the compound classes that are of interest in their sample and can therefore provide elemental compositions whose theoretical mass can be computed and used to annotate some of the grouped candidate compounds. To facilitate this process, GlycReSoft includes an optional Compound List Generator tool that allows the user to automatically generate a list of elemental compositions and masses given a set of biomolecular residues expected in the sample. The generator allows the user to specify algebraic rules for combining the biomolecular residues. Alternatively, the user may generate a custom compound list using a spreadsheet program. If a compound list is used, GlycReSoft performs a compound list matching step in an attempt to annotate as many candidate compounds as possible. Successful matching requires agreement between the masses in the theoretical compound list and the observed representative masses computed through the combination of LC/MS detection, deconvolution by Decon2LS/DeconTools, and grouping by GlycReSoft. GlycReSoft matches a candidate compound with observed representative mass *M_C_* to a known compound with theoretical mass *M_τ_* if 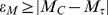 , where 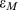 is a user-defined matching tolerance measured in ppm and should reflect the precision of the mass spectrometer (Figure 2). Choosing acceptable values for the user-defined error tolerances 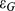 , 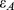 and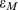 is a matter of balancing the sensitivity and specificity of the data and can only be optimized empirically. With the LC/MS data described in this work, we have found that values of 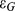 = 80 ppm, 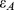 = 5 ppm and 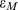 = 20 ppm provide the best balance between true positive and false positive rates using an Agilent 6520 QTOF mass spectrometer with precision of <5 ppm.

The final step in processing by GlycReSoft is to score and rank the candidate compounds, each of which represents one or more deconvoluted masses from the Decon2LS/DeconTools output following the grouping stages. GlycReSoft first summarizes relevant supporting data reported by Decon2LS/DeconTools for the grouped peaks. We refer to these peak summary statistics as features of the candidate compound groups. Features reflect quantities such as the number of charge states recovered, mean and range of elution time, absolute number of MS scans, total volume accumulated, number of adduct forms and presence of A+2 isotopic peaks. These features, described in the next section, provide the basis for scoring of candidate compounds. A compound list, if generated by the user, is used by GlycReSoft to annotate predicted compounds and to train the scoring function to recognize known compounds that represent true positives. Thus GlycReSoft provides a supervised scoring function when the user generates a compound list hypothesis. However, if no compound list is present, GlycReSoft scores compounds using an unsupervised framework.

In supervised scoring, the list of candidate compounds is first matched to the user-generated compound list hypothesis using the method described above to produce an annotated list. Candidate compounds that are annotated successfully represent positive examples and those that are unannotated represent negative examples. Next, a logistic regression is performed on the list of compounds to build a linear model, λ, based on the weighted features of each candidate compound, which is mapped onto a logistic function, 
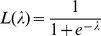
. Identification of the optimal model λ* entails learning the combination of feature weights that best separates the positive and negative examples. Annotated candidates are assigned an expected score of *L(λ) = 1* and unannotated candidates are assigned an expected score of *L(λ) = *0. The learned regression model *λ^+^* approximates λ*, where *λ^+^* is defined on the range of (0,1). For each candidate compound, *c*, the final score is computed empirically on the learned model as 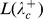.

In unsupervised scoring, the absence of a compound list hypothesis makes positive and negative examples indistinguishable and complicates the ability to learn a model. To score compounds *ab initio*, we use a method based on the sum of relative ranks. The relative rank for a candidate compound 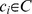 about feature 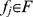 is denoted *R_ij_*, where *C* is the set of candidate compounds in the list and *F* is the set of features. *R_ij_* is defined as the ranking of *c_i_* relative to all other candidate compounds in *C* when looking at feature *f_j_* independently. *R_ij_* values are standard competition rankings (i.e., a “1, 2, 2, 4”-style ranking) computed by sorting candidate compounds by feature *f_j_*, in either ascending or descending order based on feature type. In practice, the user may find it beneficial to assign a vector of weights 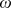 to the features during unsupervised scoring, where 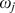 is the weight associated with feature *j*. Thus for a candidate compound *c_i_*, the total score *S* is defined as:
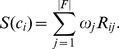



Scores are reported as a percentage of the best possible score.

### Features

GlycReSoft uses the following features for scoring. Each feature was designed to assess specific qualitative and quantitative characteristics of the raw Decon2LS/DeconTools peak groupings that define the candidate compounds.

**Figure 3:**
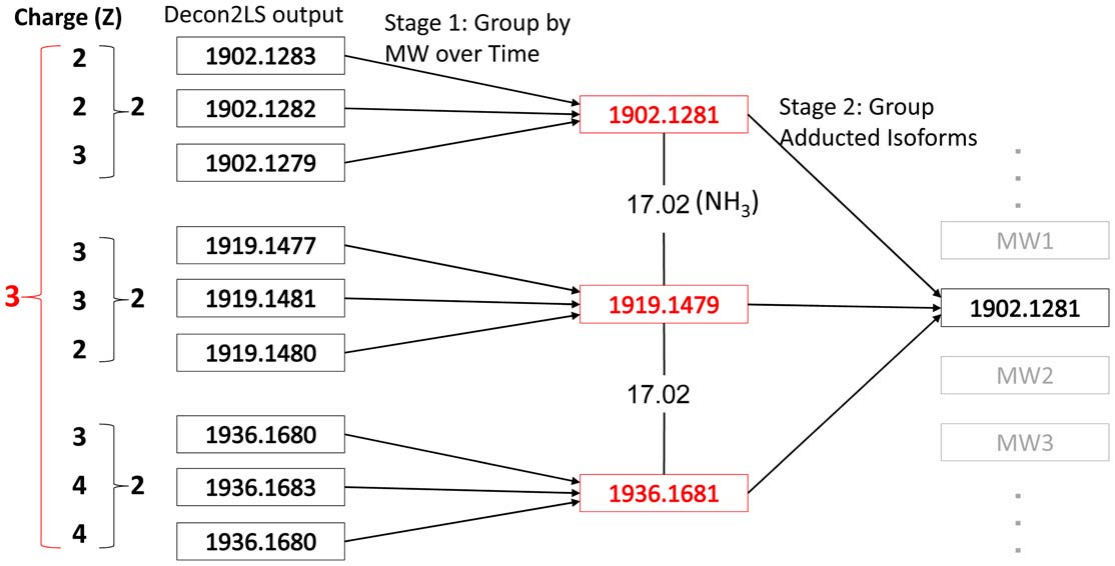
GlycReSoft raw data grouping. In this example, rows of raw Decon2LS/DeconTools output are grouped into a unique set of molecular weights, corresponding to candidate compounds.

**Figure 4:**
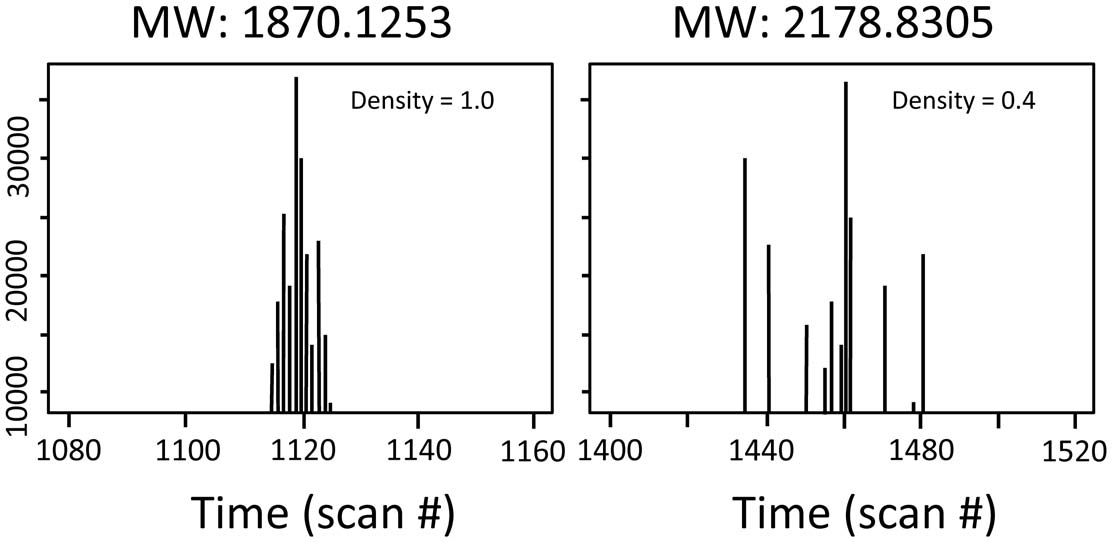
Two example candidate compounds representing a densely eluting compound (left) and a sporadically eluting compound (right).
- **Number of Modification States.** In HILIC LC/MS datasets, it is common for ammonium adducts to be observed due to the chromatographic mobile phases that contain ammonium salts [11]. To account for this, GlycReSoft searches the candidate compounds from the Decon2LS/DeconTools output in the first grouping step and groups those masses shifted by equivalents of ammonia (Figure 3). The presence of such mass shifts between deconvoluted masses was weighted in favor of a true positive identification for a given compound.
- **Total Volume.** Deconvoluted masses that correspond to noise peaks in the MS data tend to be of lower abundances than those of true positive compounds. We computed the volume (V) of deconvoluted peaks as 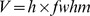, where h is the height of the peak and fwhm is the full width at half maximum of the peak. Thus the volume approximates the area of under the peak clusters shown in Figure 4.
- **Expected A: A+2 Peak Abundance Ratio Error.** The Decon2LS/DeconTools program reports the volumes of the monoisotopic peak, A, and an accumulated abundance across the corresponding isotopic cluster. Decon2LS/DeconTools also reports the abundance of the A+2 isotopic peak. If the reported mass is a true positive compound, we expect that the ratio of the abundance of the monoisotopic peak A to the abundance of the A+2 peak follows a linear distribution with respect to the MW of the candidate compound. We therefore fit a line through the distribution of the A:A+2 peak abundance ratios of the sample using a robust linear regression (Figure S1) to define the expected value. In practice, we only fit the ratios of matched compounds (in supervised learning) or ratios above zero (in unsupervised learning). We reported the error between the actual value and the expected value as an additional feature for the compound.
- **Centroid Scan Error.** The profile of the abundance of a true positive compound as a function of time approximates the chromatographic peak shape. This peak shape was approximated as a normal distribution and the centroid was used to represent the elution time of a compound in the LC/MS data. Generally, the elution time increases with compound mass. In the data analyzed, the centroid scan and the molecular weight follow a linear relation (Figure 5). We therefore reported the centroid scan error between the actual value and the expected value as an additional feature for the compound. Expected centroid scan values were computed via linear regression in the same manner as described for expected A:A+2 ratios.

- **Number of Scans.** A true positive compound elutes from the LC column at a specific range of time. Therefore, GlycReSoft computes the number of scans in which a compound was present as a feature to help distinguish it from noise.
- **Number of Charge States.** Most glycan ions are observed in multiple charge states. GlycReSoft therefore tracks the number of charge states observed for a given deconvoluted mass to provide confidence regarding the validity of a candidate compound positive identification. For example, in Figure 3, there are three charge states (z = 2, 3, and 4) for the candidate compound, some of which are observed for adducted forms.
- **Scan density.** A true positive compound is expected to elute from the LC column within a time range defined by the chromatographic peak width. GlycReSoft computes the scan density:
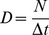
where N is the number of scans in which a mass was observed and △t is the amount of time between the first and last time point reported by this compound (see Figure 4). Thus a candidate compound that elutes continuously over a specific time window will have D> = 1, whereas a candidate compound that elutes sporadically over the entire LC/MS run will have D<<1.

**Figure 5:**
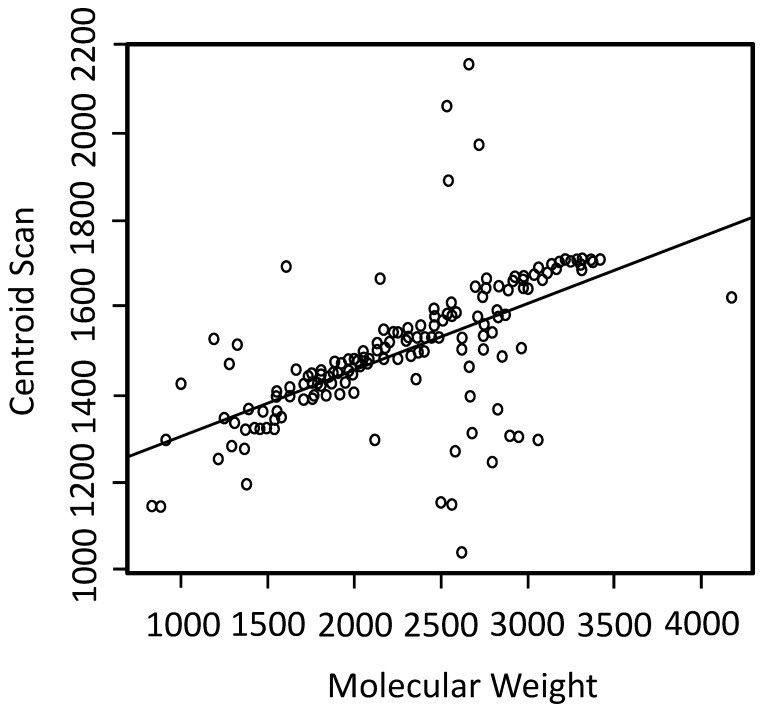
A linear fit of the centroid scan numbers (average elution time point).

**Figure 6:**
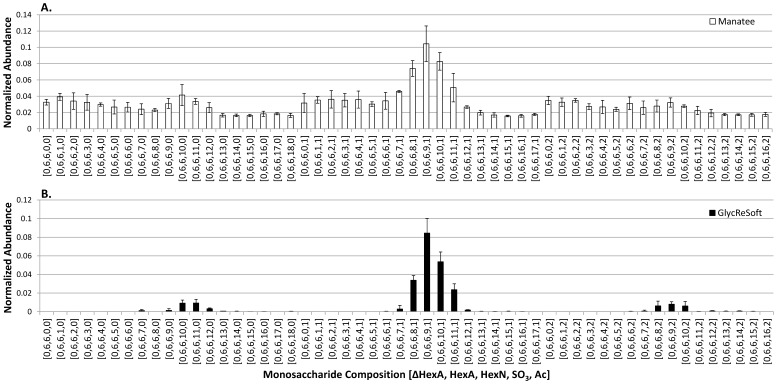
Comparison of Manatee (A) and GlycReSoft (B) for compositional profiling of HS oligosaccharides. HS oligosaccharides were prepared as described in the methods section and analyzed using negative polarity HILIC LC/MS. The histograms were normalized relative to the average of the 10 most abundant compositions. Compound compositions are given as [ΔHexA, HexA, HexN, SO_3_, Ac].
- **Average Signal-to-Noise Ratio.** For a true positive compound, the signal-to-noise ratio should be above a threshold value, and this was used as a standard to measure the quality of a deconvoluted mass peak. GlycReSoft uses the average signal-to-noise ratio of the candidate compound group as a feature.

**Figure 7:**
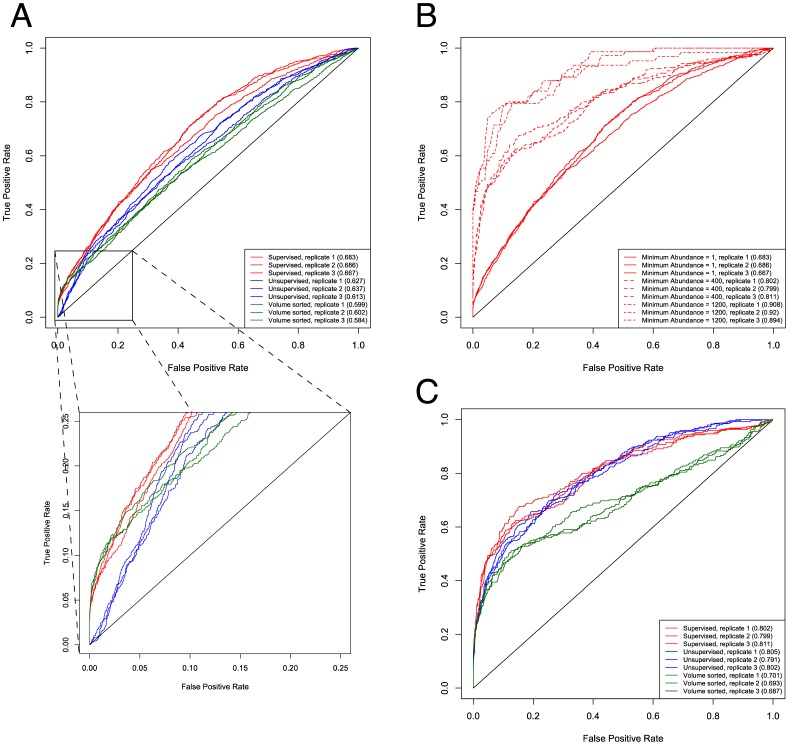
ROC curves comparing a supervised learning (red), unsupervised learning (blue) and a naïve classifier based on peak volume (green) for triplicate analysis of lung HS. ROC curves were calculated for triplicate LC/MS runs acquired using lung HS. Area under the ROC curve (ROC) is indicated in parentheses. (A) Comparison of supervised, unsupervised, and volume sorting-based scoring methods in the absence of a minimum abundance noise filtering threshold. An expanded range is shown below. (B) Supervised scoring results compared at different levels of minimum abundance thresholding, where minimum abundance of 1 is identical to (A). (C) Comparison of all scoring methods using minimum abundance threshold of 400.

### Compound List Generator

GlycReSoft includes a function that automatically generates a list of compounds and their theoretical masses, representing a hypothesis of sample composition, based on algebraic rules defined by the user. To utilize this function, the user specifies the elemental composition of the monosaccharide residues, their combination rules, and allowable adducts. The default settings include the most common animal monosaccharides (Figure S2). The user can change these residues as needed. An example of the compound list generator output is provided in Figure S3. The compound list file is used by GlycReSoft to produce the learned output. The user may also generate a compound list file of the same comma separated format using a spreadsheet program, if desired.

**Figure 8:**
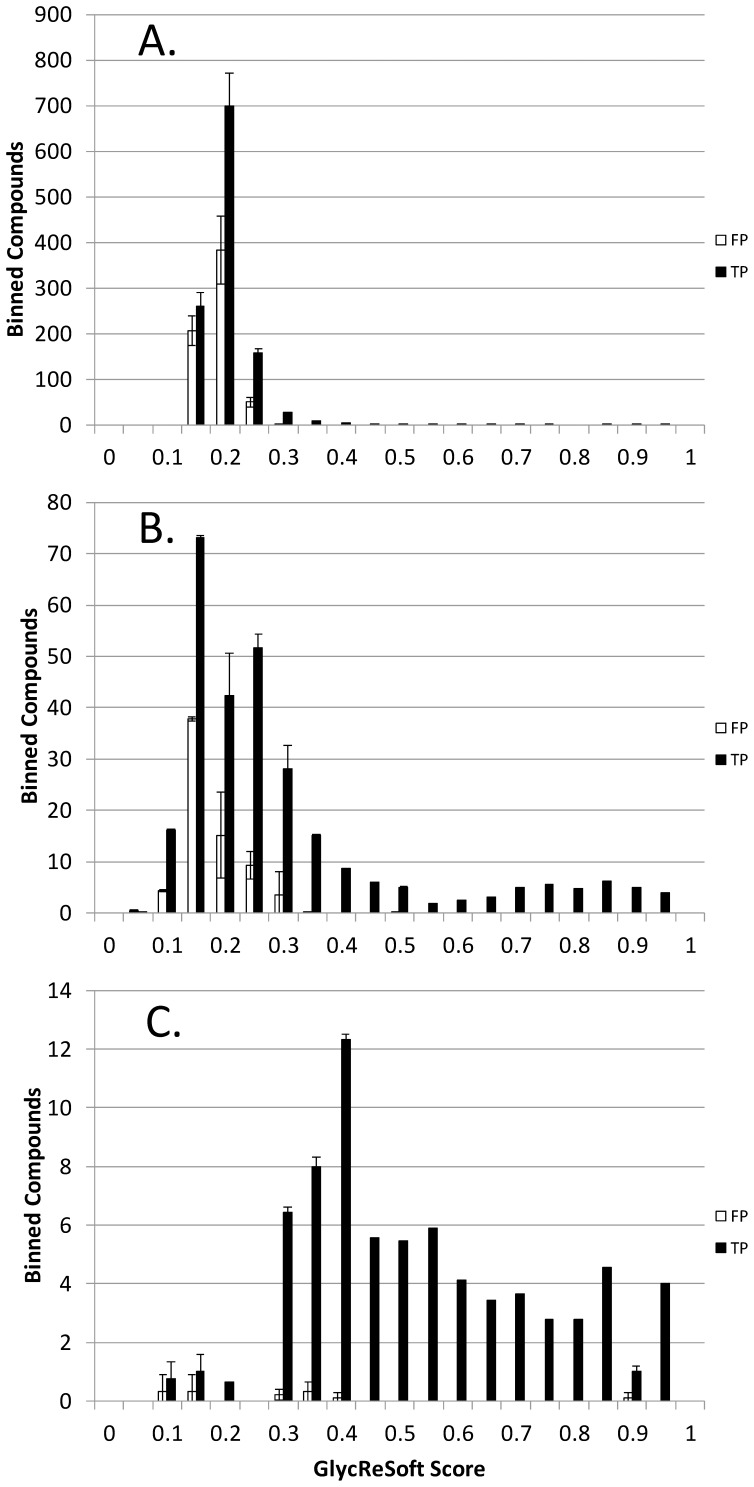
Histograms showing number of binned true positive (TP) and false positive (FP) compounds detected as a function of GlycReSoft score for three volume thresholds, (A) 1, (B) 400 and (C) 1200.

### Open Source Public Archive

The GlycReSoft program and source code has been archived publically under a GNU v. 3.0 general public license. The program is available at the following web site: Google code (http://code.google.com/p/glycresoft/downloads/list).

**Figure 9:**
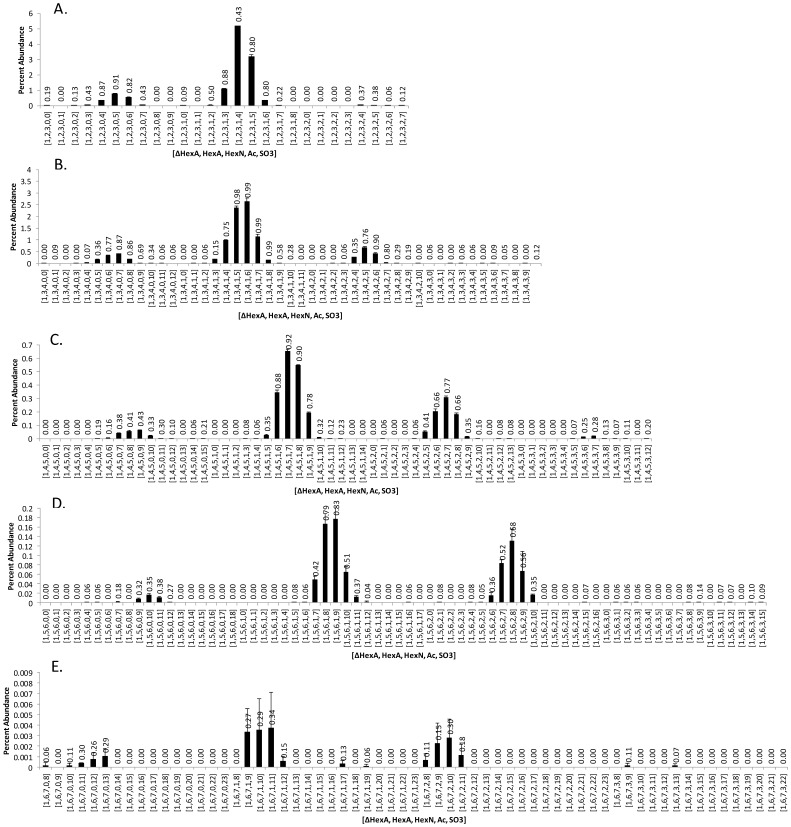
Histograms showing the compositions and percent abundances for lung HS oligosaccharides. The GlycReSoft score for each composition is labeled. Compositions were assigned using GlycReSoft minimum abundance setting of 400. Histograms were displayed with a GlycReSoft score threshold of 0.16. (A) degree of polymerization (dp) 6, (B) dp8, (C) dp 10, (D) dp 12, (E) dp14. The error bars reflect the standard deviation of the average values obtained from three LC/MS analyses.

## Results

### Application of GlycReSoft to Glycomics Profiling of Heparan Sulfate

The ability to assign glycan profiles with confidence from LC/MS profiling datasets was a primary goal for the development of GlycReSoft. For the purpose of this work, glycan profiles refers to the monosaccharide compositions and abundances determined using LC/MS data. Each composition is assumed to contain a mixture of glycan isomers. The analysis of such isomers requires additional MS and tandem MS experiments that are beyond the scope of the present version of GlycReSoft.

In earlier work by this group, a program (Manatee) was developed for extraction of abundances for targeted glycan compositions from glycomics datasets [19]. Manatee works rapidly to give users an overview of the dataset, but lacks noise reduction functions and is susceptible to high false negative and false positive rates based on the quality of the targeted compound list. False negatives are produced by an overly exclusive compound list where true compounds not in the list are ignored; effectively eliminating the ability to identify novel compounds. False positives are produced by an overly inclusive compound list; Manatee extracts ion signal for all targeted glycan compositions, and all have an abundance value >0. This is illustrated in Figure 6A, showing the abundance profile for degree of polymerization (dp)12 oligosaccharides from a bovine lung sample. At this relatively low normalized abundance of 0.1, the signal-to-noise ratio of the most abundant composition is approximately 3 and it is difficult to determine which compositions are true compounds and which are noise. Figure 6B shows the abundance profile obtained using GlycReSoft from the DeconTools output of the data. The signal-to-noise ratios are improved by more than 10-fold and this enables confident assignment of glycan compositions including those at a normalized abundance of 0.01 and below.

By producing candidate compounds empirically and independently of a compound list, GlycReSoft does not suffer from the high false positive and false negative rates inherent to targeted methods, such as Manatee. GlycReSoft allows the user to designate a score threshold below which compounds are not reported, providing a means to manually account for false positive/negative rates. The method focuses on the fact that a true positive compound should be distinguishable from noise in the LC/MS data and this separation can be automated with high precision.

#### Evaluation of GlycReSoft performance

Figure 7 compares receiver operating characteristic (ROC) plots obtained for the lung HS data for supervised, unsupervised (with all feature weights set to one) and a naïve classifier based on peak volume sorting. Area under the ROC curve (AUC) is reported in the parentheses in the figure inset. In (A), no abundance threshold is applied to filter noise from the raw DeconTools output, and it is clear that supervised learning outperforms unsupervised learning which in turn outperforms volume sorting. The expanded plot shows that volume sorting performs well for the most easily matched compounds, but produces high false positive rates below a threshold value. Unsupervised learning does not do well with the most easily matched compounds, likely because the features are unweighted. The unsupervised learning outperforms volume sorting over the full data set, indicating that features other than volume are important. Supervised learning performs equally well as volume sorting for the most easily matched compounds but continues to perform well after the volume sorting results deteriorate.

The similar performance observed between supervised scoring and volume sorting at the lowest false positive rates indicates that supervised scoring likely weights volume as the most important feature. In Figure 7B, the effect of applying GlycReSoft’s minimum abundance threshold, at values of 1 (no threshold), 400 and 1200, is shown in the context of enhancing the performance gain by filtering out noisy Decon2LS/DeconTools rows, which tend to be low in abundance. Removing such noise greatly improves the regressed model and effectively removes large amounts of false positives (see below), but also will remove some true positives of low abundance. Thus finding an abundance threshold that balances maintaining true positives and removing false positives is essential. In our data, we found a minimum abundance of 400 to be a good balance using estimates of false positive rates (see below), and the predictive performance using this minimum abundance threshold is displayed in Figure 7C.

Estimation of false positives. In the interpretation of glycan LC/MS data it is important to consider the limitations of the informatics approach. First, the solution file may not be inclusive of all glycans present in the sample. GlycReSoft addresses this issue by enabling the user to investigate unassigned MS peaks that receive a score above a threshold value. The user may then address whether the solution file should be modified in an attempt to include such peaks. Second, mass spectral peaks derived from chemical noise may be deconvoluted by Decon2LS/DeconTools and result in the false assignment of glycan compositions by GlycReSoft. The use of a target decoy database approach [21] for false positive (FP) filtering and false discovery rate (FDR) estimation has been adopted widely in the proteomics field [22], [23], [24], [25], [26], [27]. The decoy database consists of reversed or randomized protein sequences, the number of positive identifications from which is used to estimate FPs in the target database. Such decoy databases enable the user to adjust score thresholds to produce acceptable levels of FPs and FDR. In an analogous manner, we used the GlycReSoft glycan generator to produce a solution file in which the elemental compositions of the monosaccharide residues had been randomized. This randomized compound list was appended to a list generated using the same algebraic rules and the correct elemental compositions. Three such solution files were constructed using the randomized monosaccharide compositions shown in Figure S4.

Figure 8 shows a set of histograms showing the number of true and false positive compounds binned as a function of GlycReSoft score. The plot shown in (A) was produced using a minimum abundance threshold setting of 1 in the GlycReSoft parameters window. The masses that matched the true compounds in the solution were labeled as true positives (TP). Those that matched the masses of compounds corresponding to randomized glycan elemental compositions were labeled as false positives (FP). The Y axis is the total number of compounds detected in 0.05 unit GlycReSoft score increments. The histogram in (A) shows a large number of TP and FP compounds detected with GlyReSoft scores of 0.2 or below. When the GlycReSoft minimum abundance threshold was increased to 400 (B), the number of TP and FP compounds decreased approximately 10-fold. Increasing the minimum abundance threshold to 1200 (C) removed nearly all compounds below a GlycReSoft score of 0.3. From these data, a minimum abundance threshold of 400 and a GlycReSoft score of 0.16 were selected as the optimal settings for the data set since these are the values at which TP exceeds FP by a ratio of 2∶1 while maintaining reasonable sensitivity in the expected TP set.

A key capability of GlycReSoft is to provide the user with metrics for appropriate choice of analysis parameters. The histograms for lung HS oligosaccharides identified using a GlycReSoft minimum abundance of 400 and a score threshold of 0.16 are shown in Figure 9. Using these values, the GlycReSoft scores (shown for each composition on the histograms) provide a confidence metric regarding the assigned oligosaccharide compositions. Even for the lowest abundance compositions, dp 14, shown in (E), GlycReSoft scores >0.2 are obtained for most compositions >0.1 in percent abundance. By comparison, histograms obtained using a minimum abundance value of 1 show considerably more compositions with abundances >0 for the same dp 14 oligosaccharides (see Figure S5E). Consistent with the histogram shown in Figure 8A, this pattern demonstrates unacceptable levels of false positives when using a minimum abundance value of 1. Histograms obtained using a minimum abundance value of 1200 showed no compositions with percent abundances <0.01 (see Figure S6), consistent with the removal of true positives. This example demonstrates the use of the GlycReSoft output to optimize analysis parameters for general application to a larger dataset.

## Discussion

The primary bottleneck for dissemination of LC/MS glycomics profiling methods is the complexity of the data. Each glycan composition is present as multiple charge and adduct states. For the HS datasets described here, the time required for exhaustive analysis of a single dataset by manual inspection is on the order of weeks. The previously described Manatee tool allows rapid analysis of the data but is limited to relatively abundant peaks and does not provide a confidence measure. It is therefore desirable to implement a solution that includes noise reduction and confidence measures for LC/MS glycomics datasets. GlycReSoft takes the output of the publically available Decon2LS/DeconTools deconvolution program and enables the user to choose between supervised and unsupervised methods to score the output peaks. Glycomics data interpretation is driven by the chemical nature of the glycan compound classes. GlycReSoft includes a compound list generator, the output of which can be used to determine a scored output using a machine learning algorithm. All LC/MS peaks are scored, allowing the user to set threshold values for analysis. The GlycReSoft output provides metrics that enable the user to develop analysis parameters that are appropriate for the analysis of datasets containing multiple LC/MS runs. The program is open source and has been archived under a GNU 3.0 general public license.

GlycReSoft utilizes linear learning functions for scoring that have the advantage of being easily interpreted, but the disadvantage of being limited to linear relationships. Using more complex non-linear learning functions might provide better performance, but would also require logical reporting methods. We have shown that GlycReSoft greatly improves glycan recognition through automation of LC/MS profiling and by casting it as a machine learning problem. Future iterations on this theme are planned to extend GlycReSoft capabilities through the use of additional features than the ones described here. There is currently no gold standard of features to be used for scoring, and a more profound exploration of features, as well as their relationships, would be beneficial to this problem.

## Supporting Information

Figure S1 **A linear fit of the A:A+2 abundance ratio.**
(TIF)

Figure S2 **Screen shot showing the GlycReSoft composition generator. The parameters entered were used in the present publication.**
(TIF)

Figure S3 **An example showing the format for the compound list. First column is chemical formula. Second column is molecular weight, Third column is the mathematical formula representing the composition of each groups in both main chain (bracket one) and adducts (bracket two). Compositions in the first bracket are given as [ΔHexA, HexA, HexN, SO3, Ac]. The second bracket shows the number of ammonium adducts. Although only the first place in the second bracket is used at present, a total of four places are included to allow for use of additional adducts in future versions of GlycResoft.**
(TIF)

Figure S4 **List of the randomized elemental compositions used to estimate false positives and false discovery rate using GlycReSoft. The GlycReSoft generator function was used to generate three outputs using the randomized elemental compositions shown. Each list was appended to a generator ouput using the true monosaccharide compositions. The monosaccharides were combined using the algebraic rules given in the methods section.**
(TIF)

Figure S5 **Histograms showing the compositions and percent abundances for lung HS oligosaccharides. The GlycReSoft score for each composition is labeled. Compositions were assigned using GlycReSoft minimum abundance setting of 1. Histograms were displayed with a GlycReSoft score threshold of 0.16. (A) degree of polymerization (dp) 6, (B) dp8, (C) dp 10, (D) dp 12, (E) dp14. The error bars reflect the standard deviation of the average values obtained from three LC/MS analyses.**
(TIF)

Figure S6 **Histograms showing the compositions and percent abundances for lung HS oligosaccharides. The GlycReSoft score for each composition is labeled. Compositions were assigned using GlycReSoft minimum abundance setting of 1200. Histograms were displayed with a GlycReSoft score threshold of 0.16. (A) degree of polymerization (dp) 6, (B) dp8, (C) dp 10, (D) dp 12, (E) dp14. The error bars reflect the standard deviation of the average values obtained from three LC/MS analyses.**
(TIF)
