# Supplementary figures and images for: GlycReSoft: A Software Package for Automated Recognition of Glycans from LC/MS Data
Source: PLoS One. 2012 Sep 26;7(9):e45474. doi: 10.1371/journal.pone.0045474 (PMC3458864; doi:10.1371/journal.pone.0045474)

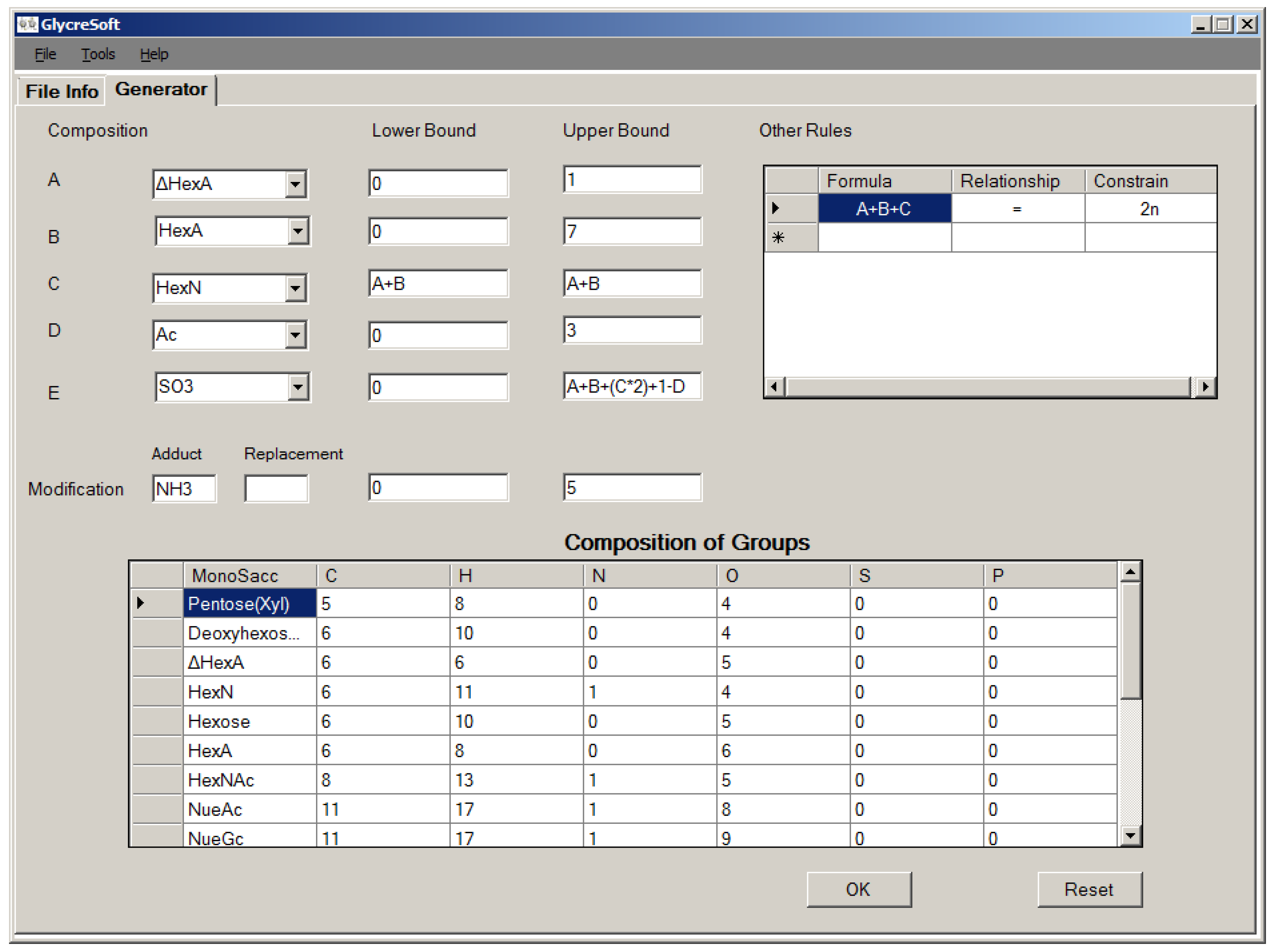

Supplement: Figure S2 — Screen shot showing the GlycReSoft composition generator. The parameters entered were used in the present publication. (TIF) [file pone.0045474.s002.tif]

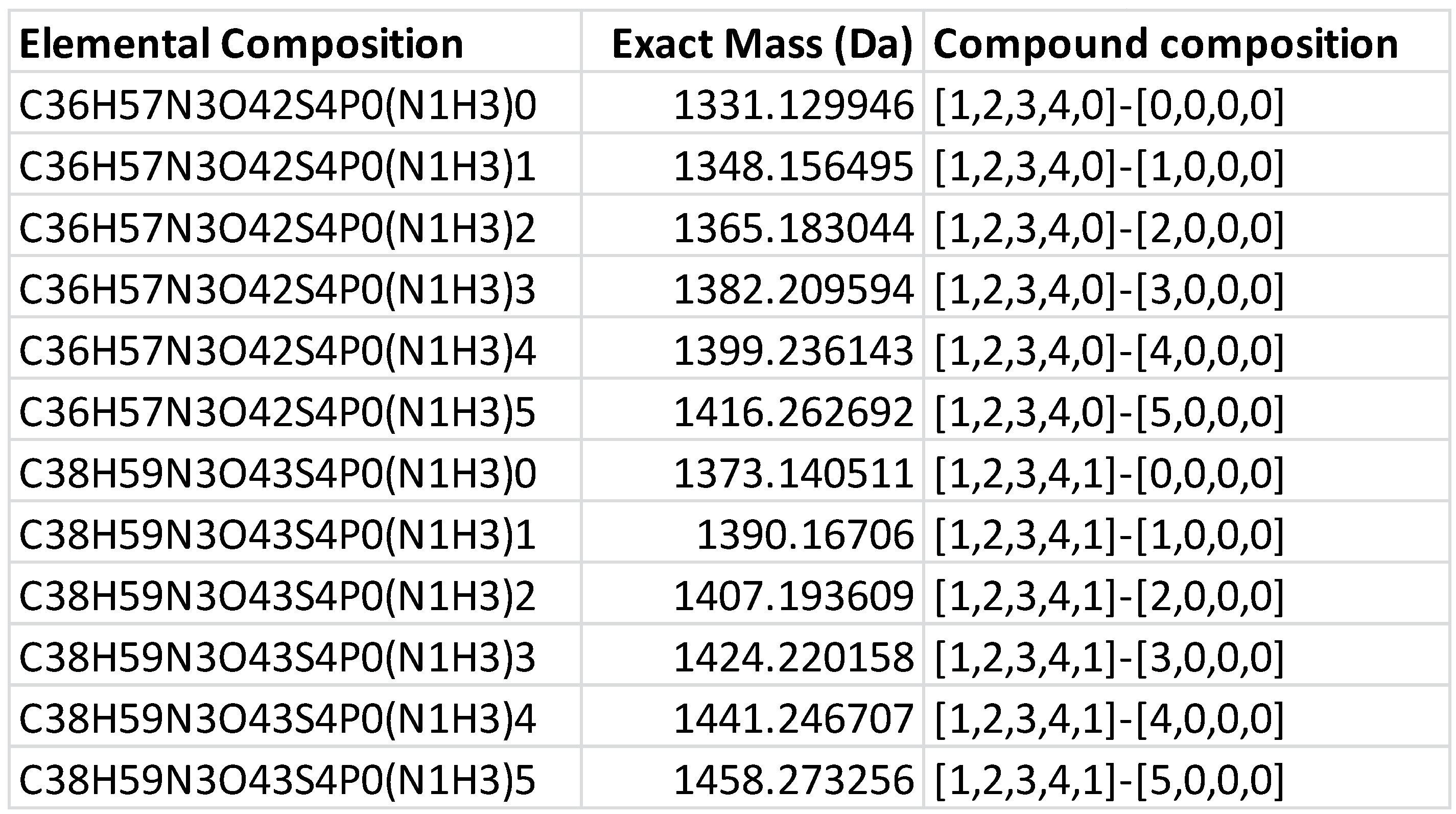

Supplement: Figure S3 — An example showing the format for the compound list. First column is chemical formula. Second column is molecular weight, Third column is the mathematical formula representing the composition of each groups in both main chain (bracket one) and adducts (bracket two). Compositions in the first bracket are given as [ΔHexA, HexA, HexN, SO3, Ac]. The second bracket shows the number of ammonium adducts. Although only the first place in the second bracket is used at present, a total of four places are included to allow for use of additional adducts in future versions of GlycResoft. (TIF) [file pone.0045474.s003.tif]

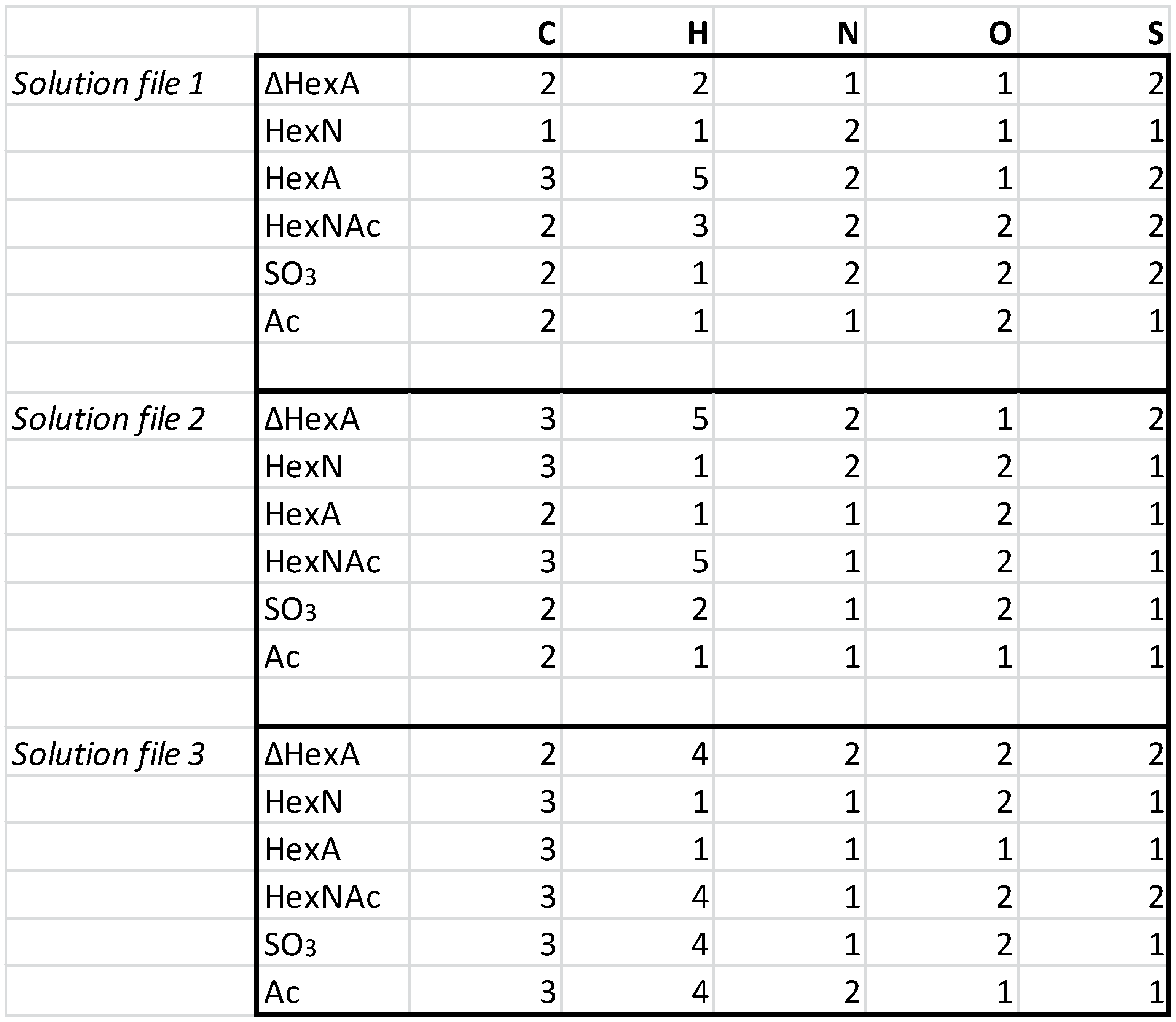

Supplement: Figure S4 — List of the randomized elemental compositions used to estimate false positives and false discovery rate using GlycReSoft. The GlycReSoft generator function was used to generate three outputs using the randomized elemental compositions shown. Each list was appended to a generator ouput using the true monosaccharide compositions. The monosaccharides were combined using the algebraic rules given in the methods section. (TIF) [file pone.0045474.s004.tif]

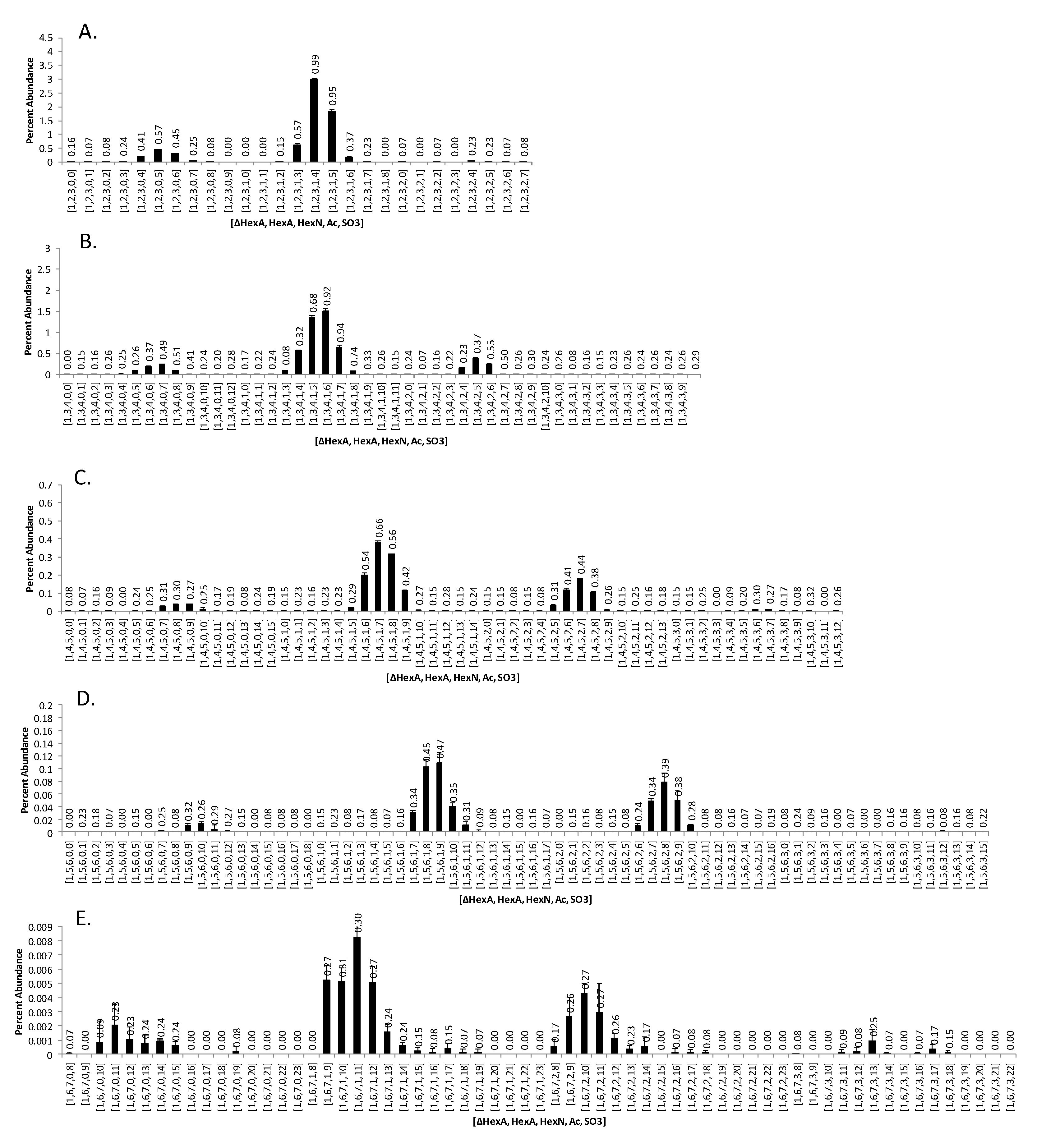

Supplement: Figure S5 — Histograms showing the compositions and percent abundances for lung HS oligosaccharides. The GlycReSoft score for each composition is labeled. Compositions were assigned using GlycReSoft minimum abundance setting of 1. Histograms were displayed with a GlycReSoft score threshold of 0.16. (A) degree of polymerization (dp) 6, (B) dp8, (C) dp 10, (D) dp 12, (E) dp14. The error bars reflect the standard deviation of the average values obtained from three LC/MS analyses. (TIF) [file pone.0045474.s005.tif]

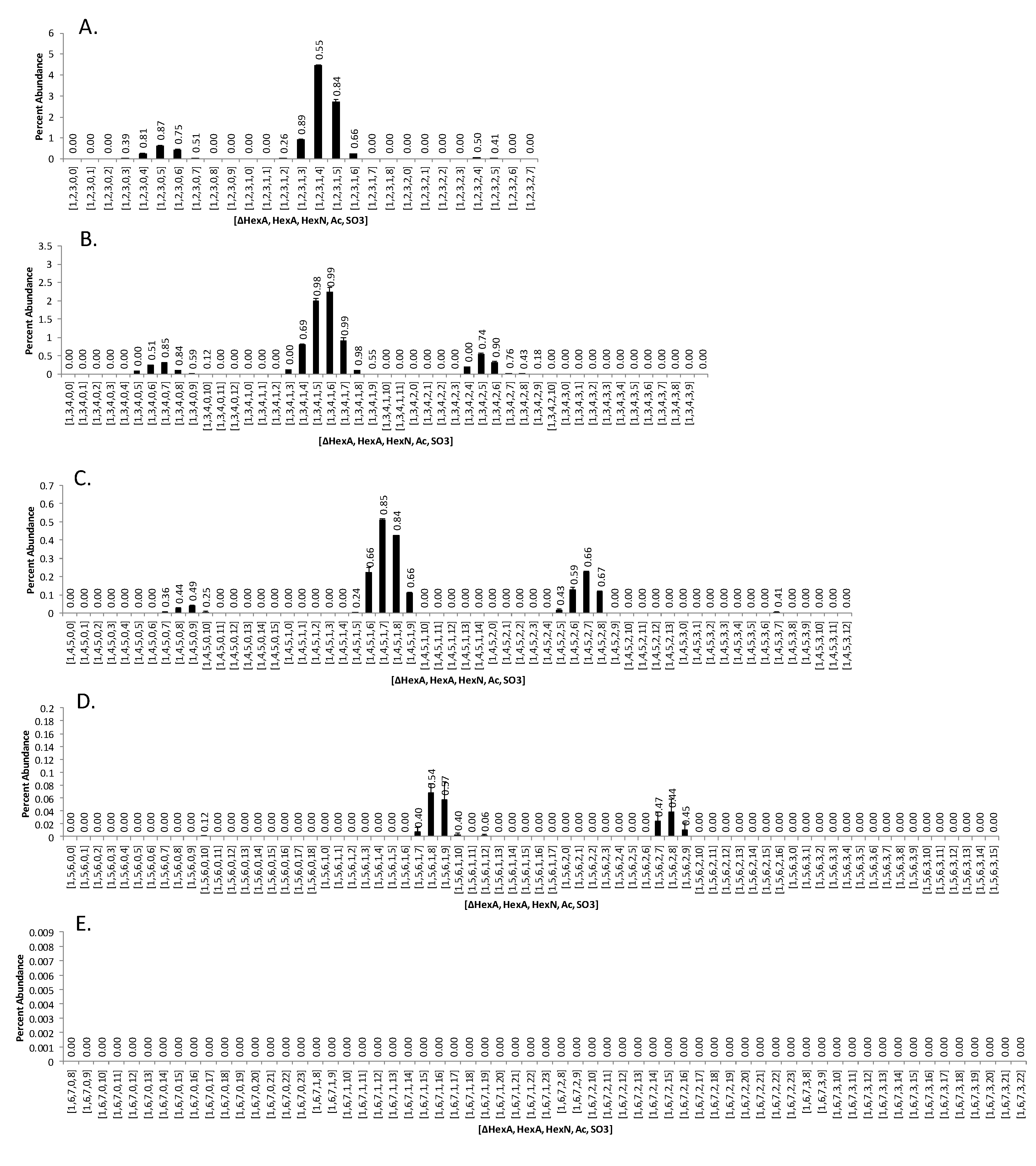

Supplement: Figure S6 — Histograms showing the compositions and percent abundances for lung HS oligosaccharides. The GlycReSoft score for each composition is labeled. Compositions were assigned using GlycReSoft minimum abundance setting of 1200. Histograms were displayed with a GlycReSoft score threshold of 0.16. (A) degree of polymerization (dp) 6, (B) dp8, (C) dp 10, (D) dp 12, (E) dp14. The error bars reflect the standard deviation of the average values obtained from three LC/MS analyses. (TIF) [file pone.0045474.s006.tif]
